# Metabolic reprogramming of the tumor immune microenvironment in ovarian cancer: A novel orientation for immunotherapy

**DOI:** 10.3389/fimmu.2022.1030831

**Published:** 2022-10-14

**Authors:** Yi Lin, Xiaoting Zhou, Yanghong Ni, Xia Zhao, Xiao Liang

**Affiliations:** Department of Gynecology and Obstetrics, Key Laboratory of Obstetrics & Gynecologic and Pediatric Diseases and Birth Defects of Ministry of Education, Development and Related Diseases of Women and Children Key Laboratory of Sichuan Province, West China Second Hospital, Sichuan University, Chengdu, China

**Keywords:** ovarian cancer, tumor immune microenvironment, metabolic reprogramming, metabolism, immunotherapy

## Abstract

Ovarian cancer is the most lethal gynecologic tumor, with the highest mortality rate. Numerous studies have been conducted on the treatment of ovarian cancer in the hopes of improving therapeutic outcomes. Immune cells have been revealed to play a dual function in the development of ovarian cancer, acting as both tumor promoters and tumor suppressors. Increasingly, the tumor immune microenvironment (TIME) has been proposed and confirmed to play a unique role in tumor development and treatment by altering immunosuppressive and cytotoxic responses in the vicinity of tumor cells through metabolic reprogramming. Furthermore, studies of immunometabolism have provided new insights into the understanding of the TIME. Targeting or activating metabolic processes of the TIME has the potential to be an antitumor therapy modality. In this review, we summarize the composition of the TIME of ovarian cancer and its metabolic reprogramming, its relationship with drug resistance in ovarian cancer, and recent research advances in immunotherapy.

## 1 Introduction

Ovarian cancer (OC) is the most lethal gynecological neoplasm due to its high mortality (1–3). Due to inadequate early detection methods and few early symptoms, ovarian cancer is the most challenging disease to identify in the female reproductive system (4). The majority of patients are frequently identified at an advanced stage, while therapeutic interventions are relatively limited. Only approximately 16% of patients are diagnosed at an early stage (FIGO I stage) and have a greater possibility of long-term survival (5, 6). The treatment of OC varies depending on its stage and classification (7). OC has an epithelial origin in more than 90% of cases, and high-grade serous ovarian cancer (HGSOC) makes up 70% of these cases (1, 8). According to the National Comprehensive Cancer Network (NCCN) Guidelines, debulking surgery combined with platinum-based chemotherapy is currently the first line of treatment for OC (7). The mortality of OC has decreased globally over the past decade as a result of lifestyle modifications and technological advancements in treatment (9). However, research has shown that early screening does not appear to reduce mortality from OC (10). More frustratingly, regardless of the type of treatment used, approximately 80% of cases of advanced ovarian cancer eventually recur and result in the progression of the disease and death (11). Given these findings, it is imperative to develop novel and reliable therapies and prevent recurrence.

Immune cells are a cluster of different cells that have differentiated from bone marrow hematopoietic stem cells (12). In tumors, immune cells have a dual role, either killing or promoting them (13). Immune cells can even become accomplices of tumor cells in the event of immune escape (14). In this regard, Stephen Paget put forth the well-known “seed and soil” hypothesis, according to which the environment in which a tumor develops determines how that tumor expresses its phenotype (15). The tumor immune microenvironment (TIME) refers to the immune infiltrative microenvironment, which consists of a large number of immune cells clustered within and around the tumor (16). With the advent of high-throughput and single-cell sequencing, research on the TIME has been fueled.

Immunotherapy is the next generation of treatment that is rapidly developing after traditional treatments such as surgery, radiotherapy, and chemotherapy (17). It functions by activating the immune defenses of the human body to eliminate tumor cells (17). A seemingly endless stream of cancer immunotherapies derived from tumor immunity have been developed, including immune checkpoint inhibitors (ICIs), adoptive cellular immunotherapy, tumor vaccines and others (17–19). Among these, ICIs have made significant progress and demonstrated excellent antitumor activity in gynecological cancer (20). However, not all patients with OC are candidates for immunotherapy, and only a small percentage of them will benefit (21, 22). Due to its cool tumor nature, OC has few infiltrating lymphocytes and responds poorly to immunotherapy (22, 23). Indubitably, OC is an immunogenic disease, and its immunogenicity is solely dependent on approximately 13% of CD8^+^ tumor-infiltrating T cells with a high affinity for antigens (24). Most likely, the underlying cause of drug resistance and treatment failure is metabolic reprogramming (25). Metabolic reprogramming, a hallmark of cancer, is the reprogramming of specific metabolic pathways inside and outside the cell to meet the demands of rapid proliferation by affecting gene expression, cellular state, and the tumor microenvironment (26). It may be crucial to investigate metabolic reprogramming of the TIME to increase therapeutic effectiveness and improve drug resistance in OC. This review focuses on the TIME in OC with an emphasis on its metabolic reprogramming and concludes with immunotherapy related to TIME metabolic reprogramming, with the goal of providing a new vision for immunotherapy in OC.

## 2 Overview of TIME metabolism in ovarian cancer

Tumors have a complicated metabolic pattern that is a highly adaptable response to hypoxia and nutritional deficiency (25). They have the ability to select the optimal metabolic phenotype based on the microenvironment. The metabolic overview of the ovarian cancer TIME is a network of substance exchange incorporating biochemical reactions. The metabolism of both tumor cells and immune cells is briefly discussed next.

### 2.1 Metabolism of ovarian cancer cells

**Figure 1:**
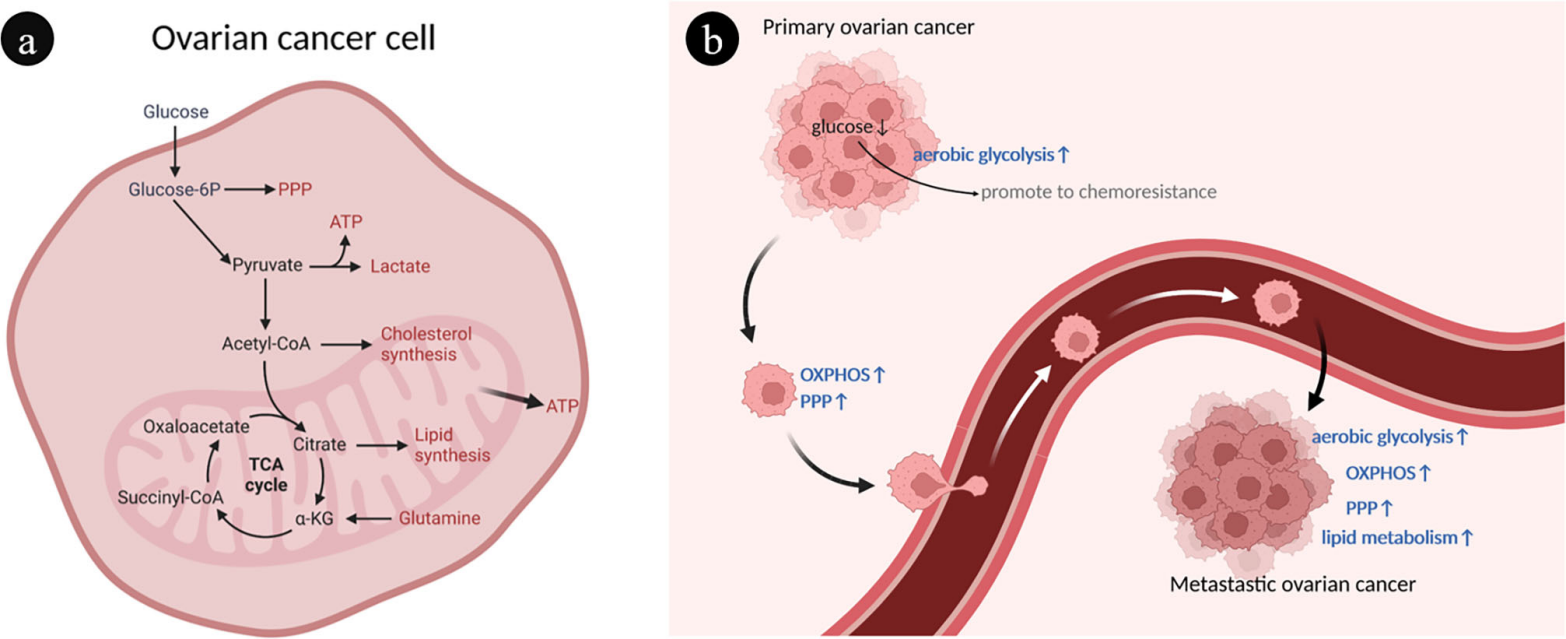
Overview of ovarian cancer cell metabolism. **(A)** Interaction of different metabolic pathways within ovarian cancer cells. There are connections between different metabolisms through metabolites. **(B)** The heterogeneity of ovarian cancer metabolism is characterized by abnormal glucose and lipid metabolism. During the development of ovarian cancer, different metabolic phenotypes are gradually acquired, such as enhanced OXPHOS, PPP and FAO. ATP, Adenosine triphosphate; FAO, Fatty acid oxidation; OXPHOS, Oxidative phosphorylation; PPP, Pentose phosphate pathway; TCA, Tricarboxylic acid.

Ovarian cancer cells have a heterogeneous metabolism, which means they are able to modify their metabolic patterns in response to their microenvironment (**Figure 1A**). The metabolic characteristics of ovarian cancer cells can be described as prominent glucose metabolism and lipid metabolism (**Figure 1B**).

The selection of aerobic glycolysis and oxidative phosphorylation as energy sources is a marked manifestation of the heterogeneity of ovarian cancer (27). Through aerobic glycolysis, often known as the Warburg effect, ovarian cancer cells primarily consume glucose to obtain energy (28). Ovarian cancer tumor cells use a substantial quantity of glucose during this process, which is finally generated and expelled as lactic acid, resulting in a localized hypoxic, hypoglycemic, and acidic microenvironment (29). The local low-glucose environment contributes to the expansion of chemical resistance to paclitaxel in ovarian cancer cells, creating a vicious cycle (30). Mitochondrial oxidative phosphorylation (OXPHOS) and the pentose phosphate pathway (PPP) were upregulated in ovarian cancer cells with metastatic capacity (31, 32). Glutamate is used by ovarian cancer cells in mitochondrial OXPHOS to produce adenosine triphosphate (ATP) and other biomolecules necessary for aggressive expansion and migration (33). Platinum-based chemotherapy increases mitochondrial OXPHOS activity of ovarian cancer cells but contributes to cancer stem cell enrichment (34).

Another characteristic of ovarian cancer cells is abnormal active lipid metabolism, which is intimately associated with tumor progression and metastasis (35). The preferential metastasis of ovarian cancer to lipid-rich tissues such as the omentum suggests that adipocyte-derived fatty acids are a significant source of energy for the rapid spreading and metastasis of ovarian cancer cells (36, 37).

### 2.2 Metabolism of immune cells in ovarian cancer

TIME has tissue heterogeneity, which means that the type and function of immune cells entering the microenvironment in various cancer types differs substantially. There is a certain gap between the resting and activated states of immune cells in ovarian cancer, as well as between different stages of tumor development. Tumor-infiltrating immune cells are an essential component of the TIME and can be classified as pro-tumor cells (tumor-associated macrophages and myeloid-derived suppressor cells) and antitumor cells (such as effector T cells and natural killer cells). The proportion and distribution of immune cells are crucial for the development and spread of tumors. **Table 1** provides a summary of immune cell metabolic propensity in the ovarian cancer TIME.

**Table 1:** Main metabolic tendencies of immune cells in ovarian cancer.

Cell type	Main metabolic pattern	Function	Refs
Immune activation			
NK cell	Aerobic glycolysis	Cytotoxicity and immunomodulation	(38, 39)
M1-TAM	Glycolysis, PPP	Inflammatory	(40, 41)
Teff	Aerobic glycolysis	Cytotoxicity	(42)
Tmem	OXPHOS	Cytotoxicity and proliferation	(42, 43)
DC	Aerobic glycolysis	Antigen presentation	(44, 45)
Immune suppression			
Treg	OXPHOS	Immune suppression and tolerance	(46)
M2-TAM	OXPHOS	Invasion Metastasis	(40, 41, 47)
MDSC	OXPHOS, FAO, Argnine metabolism	Suppression of the immune response	(48–51)

OXPHOS, Oxidative phosphorylation; PPP, pentose phosphate pathway; TAM, Tumor-associated macrophage; Teff, effector T cell; Tmem, memory T cell; Treg, Regulatory T cell; MDSC,Myeloid-derived suppressor cell; NK, Natural killer.

#### 2.2.1 Natural killer (NK) cells

Natural killer (NK) cells can be cytotoxic as well as immunomodulatory (38, 39). The primary mechanism by which NK cells attack ovarian cancer cells is antibody-dependent cellular cytotoxicity (52). The activation of NK cells depends on aerobic glycolysis (53, 54).

#### 2.2.2 Tumor-associated macrophages (TAMs)

Tumor-associated macrophages (TAMs) are significantly infiltrated in ovarian cancer, and the extent of their polarization is tightly tied to the local microenvironment (55). Under different conditions, TAMs can polarize into two types: M1-TAMs (proinflammatory/antitumor) and M2-TAMs (anti-inflammatory/protumor) (56). The expression of both M1 and M2 markers was elevated in ovarian cancer according to genome-wide expression profiling of TAMs (57). From this, it may be inferred that the monocyte phenotype of ovarian cancer is mixed polarization. The aggressiveness of ovarian cancer is directly related to the degree of infiltration of M2-TAMs. In ovarian cancer, a high M2/M1 ratio indicates a poor prognosis (58). Typically, M1-TAMs rely on aerobic glycolysis, whereas M2-TAMs are more dependent on OXPHOS (40, 41).

#### 2.2.3 T cells

T cells can be classified into a number of subtypes based on their varied roles, and these subtypes also differ in their corresponding metabolic patterns. When naive T cells differentiate into effector T cells (Teffs) in tumors, they switch from relying mostly on OXPHOS for energy to relying primarily on glycolysis (42). Memory T cells (Tmems) show high OXPHOS levels (42, 43). In particular, regulatory T lymphocytes (Tregs) have an immunosuppressive function in tumors, where their recruitment contributes to the immune escape of ovarian cancer (59). Tregs have higher levels of glucose uptake and glycolysis because their genes associated with glucose metabolism are substantially expressed (46). A high glycolytic level of CD4^+^ Tregs was likewise found in coculture with SKOV3 ovarian cancer cells (46). In addition, Tregs can also rely on OXPHOS produced by fatty acid oxidation (FAO) to provide energy (60).

#### 2.2.4 Dendritic cells (DCs)

Dendritic cells are antigen-presenting cells that act as a bridge between innate and cellular immunity. Resting immature DCs primarily utilize mitochondrial β-oxidation of lipids and OXPHOS for energy (44). Glycolytic reprogramming takes place when DCs are activated (44). Early glycolytic reprogramming is supported by glycogen metabolism in DCs (45). The TIME inhibits the function of DCs in malignancies. In the ID8 ovarian cancer mouse model, tumor cells led to overexpression of suppressor of cytokine signaling 3 (SOCS3) in DCs and lowered the activity of the corresponding pyruvate kinase (a crucial enzyme for glycolysis), which inhibited the function of DCs (61).

#### 2.2.5 Myeloid-derived suppressor cells (MDSCs)

A group of immature heterogeneous cells from the bone marrow known as myeloid-derived suppressor cells (MDSCs) have immunosuppressive properties (62). It has been proven that enhanced lipid uptake and FAO by MDSCs activate immunosuppressive mechanisms (48). OXPHOS (fueled by glutamine) and tricarboxylic acid (TCA) cycling were elevated in MDSCs, which were demonstrated to have a high-energy metabolic profile in the ID8 ovarian cancer mouse model (49). Additionally, arginine metabolism is of great importance in MDSCs. MDSCs can regulate T cell function by removing essential metabolites such as arginine from the microenvironment (50). MDSCs were found to be dependent on arginase-1 (ARG1) to exert their T cell immunosuppressive phenotype (51).

The metabolism of immune cells in ovarian cancer is heterogeneous. Immune-activated cells are more dependent on anaerobic glycolysis, whereas immunosuppressive cells tend to use multiple metabolic pathways, including OXPHOS, to meet their energy needs. Modulation and intervention of immune cell metabolic patterns can aid in breaking the immunosuppressive TIME of ovarian cancer.

### 2.3 Overall metabolism of ovarian cancer patients

Compared to healthy individuals, patients with OC have a generally altered metabolism. The analysis of serum metabolomic data from epithelial ovarian cancer (EOC) revealed considerable heterogeneity in the metabolism of distinct subtypes of ovarian cancer (63). This demonstrates the metabolic heterogeneity of various subtypes in ovarian cancer. For instance, Hishinuma, E., et al. found an increase in tryptophan and its metabolite kynurenine by a wide-target metabolomic analysis of plasma from patients with epithelial ovarian cancer, and a larger ratio in its proportion was associated with a worse prognosis (57). The metabolism of ovarian clear cell carcinoma (OCCC), on the other hand, shows increased expression of glycolysis and oxidative stress genes, as well as improved mitochondrial OXPHOS and glycolysis (64). Additionally, the oncogene ARID1A has loss-of-function mutations in more than 50% of OCCC patients, which leads to decreased glutaminase (GLS) inhibition (65). This consequently contributes to a particular metabolic need for glutamine in OCCC.

Furthermore, metabolism differs between stages of ovarian cancer. Altered metabolism occurs even in the early stages of ovarian cancer. High-accuracy metabolomics detection of early-stage ovarian cancer indicated increased manufacture of fatty acids and cholesterol, as well as increased expression of enzymes that block FAO (66).

## 3 Metabolic reprogramming of the TIME in ovarian cancer based on various factors

How the metabolic reprogramming of the TIME in ovarian cancer occurs is one of the hot topics of research, and multiple factors are now known to influence it. Tumor-derived cytokines, chemokines, and even the metabolic microenvironment (pH, oxygen levels, and nutrition) can have an impact on the metabolism and function of immune cells (29). Metabolic reprogramming is used to carry out the procedure. Here, we will summarize the metabolic reprogramming of the TIME in ovarian cancer in terms of different common metabolic pathways. Finally, the effect of metabolic signaling pathways on TIME reprogramming is added.

### 3.1 Glucose metabolism

Lactate is a necessary byproduct of glycolysis, and tumor cells in the TIME are extremely dependent on glycolytic capacity, excreting large amounts of lactate, resulting in a localized low glucose, low oxygen, and acidic environment. Kumagai, S., et al. found that in a highly glycolytic tumor microenvironment, Tregs actively take up lactate through monocarboxylate cotransporter 1 (MCT1) and enhance the expression of programmed death receptor 1 (PD-1) by promoting NFAT1 entry into the nucleus (67). However, PD-1 expression was reduced in Teffs (67). As a result, PD-1 treatment was doomed to fail. Furthermore, increased lactate in ovarian cancer promotes the production of VEGF, a potent inducer of angiogenesis in tumors (68). The ensuing cellular invasion is a critical element in the proliferation and metastasis of ovarian cancer. In conjunction with GM-CSF and M-CSF, tumor-derived lactic acidosis in ovarian cancer was discovered to drive macrophage differentiation into a preinflammatory tumor phenotype (VEGF^high^ CXCL8^+^ IL1β^+^) (69). Furthermore, sphingosine kinase-1 in A2780 ovarian cancer cells is involved in the induction of aerobic glycolysis (70). This was demonstrated by an increase in lactate levels and expression of the MCT1, as well as a decrease in TCA cycle intermediate accumulation and the production of carbon dioxide (70). A recent study by I. Elia et al. found that tumor-derived lactate could redirect glucose metabolism in CD8^+^ T cells. Specifically, it induced a shift in pyruvate carboxylase activity to pyruvate dehydrogenase (a key enzyme of gluconeogenesis) catalytic reaction, leading to a reduction in the back-supplementation pathway of the TCA cycle, thereby suppressing tumor immunity (71).

Several enzymes involved in glucose metabolism have been implicated in the development of the malignant phenotype of ovarian cancer. Pyruvate kinase is the glycolysis rate-limiting enzyme that catalyzes the conversion of phosphoenolpyruvate and ADP to pyruvate and ATP (72). M-type pyruvate kinase is supportive of anabolic metabolism in cancers, with PKM1 and PKM2 isoforms. Normally proliferating cells are dominated by the high-activity tetrameric form of PKM2, while the low-activity dimeric form is primarily seen in cancer cells (72). TBC1D8 binds to PKM2 *via* its Rab-GAP TBC domain in invasive ovarian cancer cells, preventing PKM2 tetramerization (73). This leads to a decrease in pyruvate kinase activity, promotes aerobic glycolysis and nuclear translocation of PKM2, and induces activation of glucose metabolism and cell cycle-related genes (73). In contrast, SOCS3, which has been shown to be increased in DCs by tumor-derived substances, interacts with PKM2, resulting in decreased ATP generation under hypoxic conditions and a profound effect on the function of DCs (61). Follicle-stimulating hormone (FSH) was found to upregulate the expression of PKM2 and glycolysis in SKOV3 and OVCAR3 ovarian cancer cells in an *in vitro* assay (74).

Glyceraldehyde-3-phosphate dehydrogenase (GAPDH), a glycolytic enzyme, controls IFN-γ production in Teffs by binding to AU-rich elements within the 3’ UTR of IFN-γ mRNA (75). As a result, the ability of activated Teffs to produce IFN-γ is severely hampered when they are prohibited from participating in glycolysis even under suitable conditions (75).

**Figure 2:**
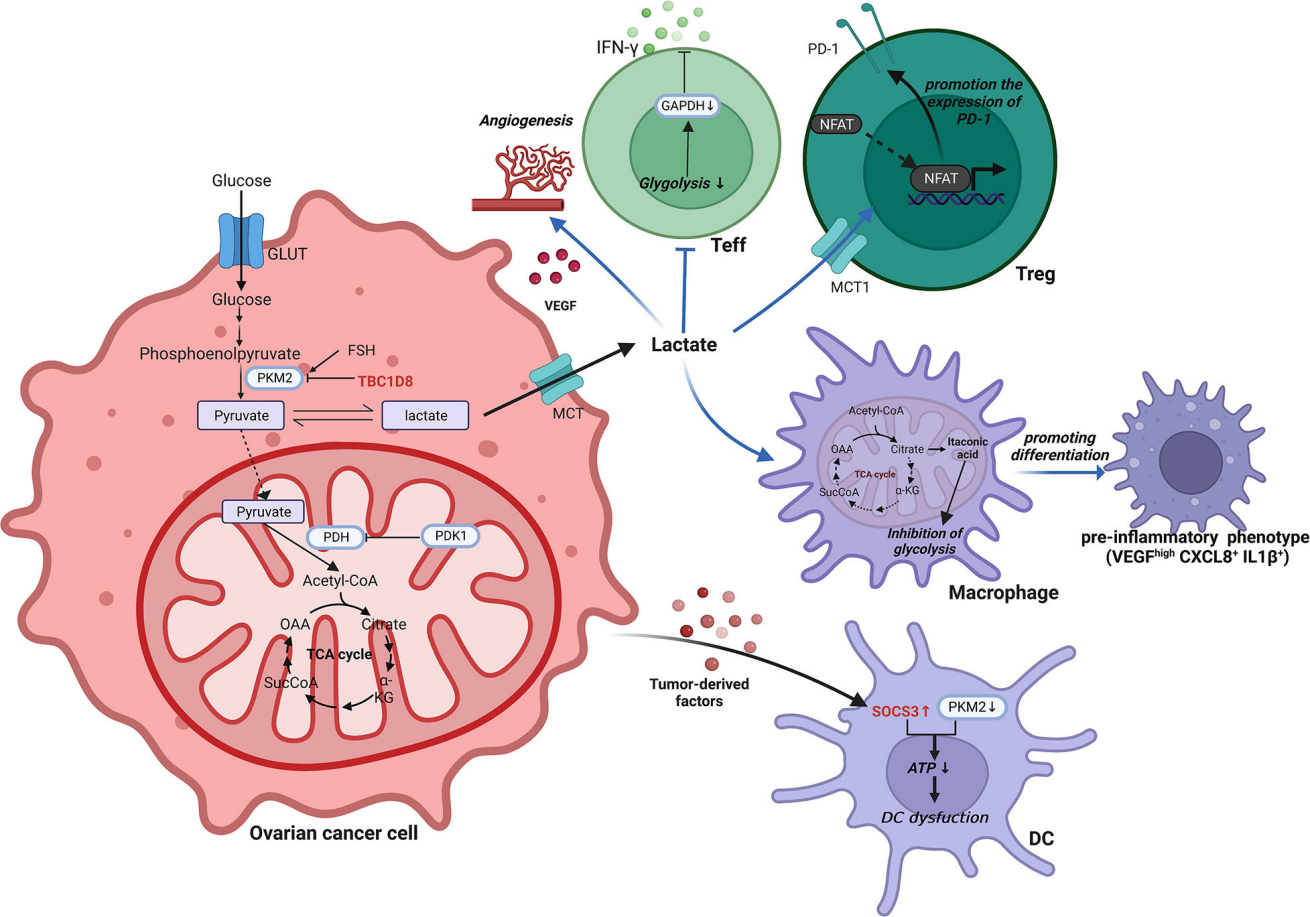
Reprogramming of glycolytic metabolism in the TIME of ovarian cancer. In the TIME of ovarian cancer, the reprogramming of glycolytic metabolism occurs in cells such as ovarian cancer cells, T cells, macrophages and DCs, resulting in a suppressive immune microenvironment that facilitates tumorigenesis and progression. ATP, Adenosine triphosphate; CXCL, C-X-C motif chemokine ligand; DC, Dendritic cell; FSH, Follicle-stimulating hormone; GAPDH, Glyceraldehyde-3-phosphate dehydrogenase; GLUT, Glucose transporter; IL, Interleukin; INF-γ, interferon-γ; MCT, Monocarboxylate cotransporter; NFAT, Nuclear factor of activated T cells; OAA, Oxalacetate; PDH, pyruvate dehydrogenase; PDK1, Pyruvate dehydrogenase kinase 1; PD-1, Programmed death receptor 1; PKM2, Pyruvate kinase M2; SOCS3, Cytokine signaling 3; TBC1D8, TBC1 domain family member 8; TCA, Tricarboxylic acid; Teff, effector T cell; Treg, Regulatory T cell; VEGF, Vascular endothelial growth factor.

In mitochondria, pyruvate is decarboxylated by pyruvate dehydrogenase kinase 1 (PDK1) to generate acetyl coenzyme A (76). It is heavily expressed in ovarian cancer and serves as a crucial hub between glycolysis and the TCA cycle (77). Inhibition of PDK1 could reverse the Warburg effect by switching cytoplasmic glucose metabolism to mitochondrial OXPHOS (78, 79). In ovarian cancer cells, abnormal elevation of PDK1 can upregulate programmed death ligand-1 (PD-L1) expression and induce increased apoptosis of CD8^+^ T cells, ultimately impairing T cell immune function (80). Pyruvate dehydrogenase kinase 2 (PDK2) is favorably connected with the prognosis of OCCC, and increased PDK2 expression decreases apoptosis, resulting in cisplatin resistance in OCCC (81). By increasing the production of reactive oxygen species although mitochondrial metabolism, PDK2 inhibition can synergistically boost sensitivity to cisplatin (81). Another classical metabolic reprogramming occurs in the TCA cycle of macrophages. Macrophages are transferred from the TCA cycle and transform cis-aconitate to itaconic acid by the activity of aconitic acid decarboxylase 1 in the TIME of ovarian cancer (82). Itaconic acid, a metabolite with anti-inflammatory activity, can exert an inhibitory effect on glycolysis (83). A brief schematic of the reprogramming of glycolytic metabolism in ovarian cancer TIME is shown in **Figure 2**.

### 3.2 Lipid metabolism

Reprogramming of ovarian cancer cells toward lipid metabolism is the initiating step for metastasis to the peritoneal cavity (84). Ovarian cancer cells can take up exogenous fatty acids (FAs) to promote dissemination in the peritoneal cavity. CD36 (FA receptor) is upregulated in metastatic human ovarian tumors (85). It was shown that ovarian cancer cells that were cocultured with primary human omental adipocytes underwent lipid metabolic reprogramming in the plasma membrane and expressed high levels of CD36 (85). Lipidomic analysis demonstrated that the high levels of polyunsaturated fatty acids, particularly linoleic acid, in ovarian cancer contributed to the tumor-promoting activity of TAMs as an efficient PPARβ/δ agonist (86).

Lysophosphatidic acid (LPA) is an important intermediate in glycerophospholipid metabolism. Through the transcriptional activation of VEGF, stimulation of Fas translocation, and other mechanisms, LPA in ovarian cancer ascites can promote tumor invasion, metastasis, and immune evasion (87, 88). TAMs were found to be a significant source of LPA in ovarian cancer by metabolomics (89). In T lymphoma cells, LPA was found to mediate apoptosis and glucose metabolism, supporting tumor cell survival (90). The corresponding metabolic regulation has yet to be demonstrated in ovarian cancer.

Prostaglandin E-2 (PGE2) is a hormone-like lipid metabolite generated by arachidonic acid *via* cyclooxygenase catalysis (91). Ovarian cancer can enhance proliferation and invasion through the PGE2/nuclear factor-kappa B signaling pathway (92). At the same time, tumor-derived PGE2 controls the production of CXCL12 and CXCR4, thereby inducing the migration of MDSCs to ascites (93, 94). In contrast, MDSC-derived PGE2 not only increases the stem cell-like properties of EOC but also increases PD-L1 expression in tumor cells (95).

### 3.3 Amino acid metabolism

**Figure 3:**
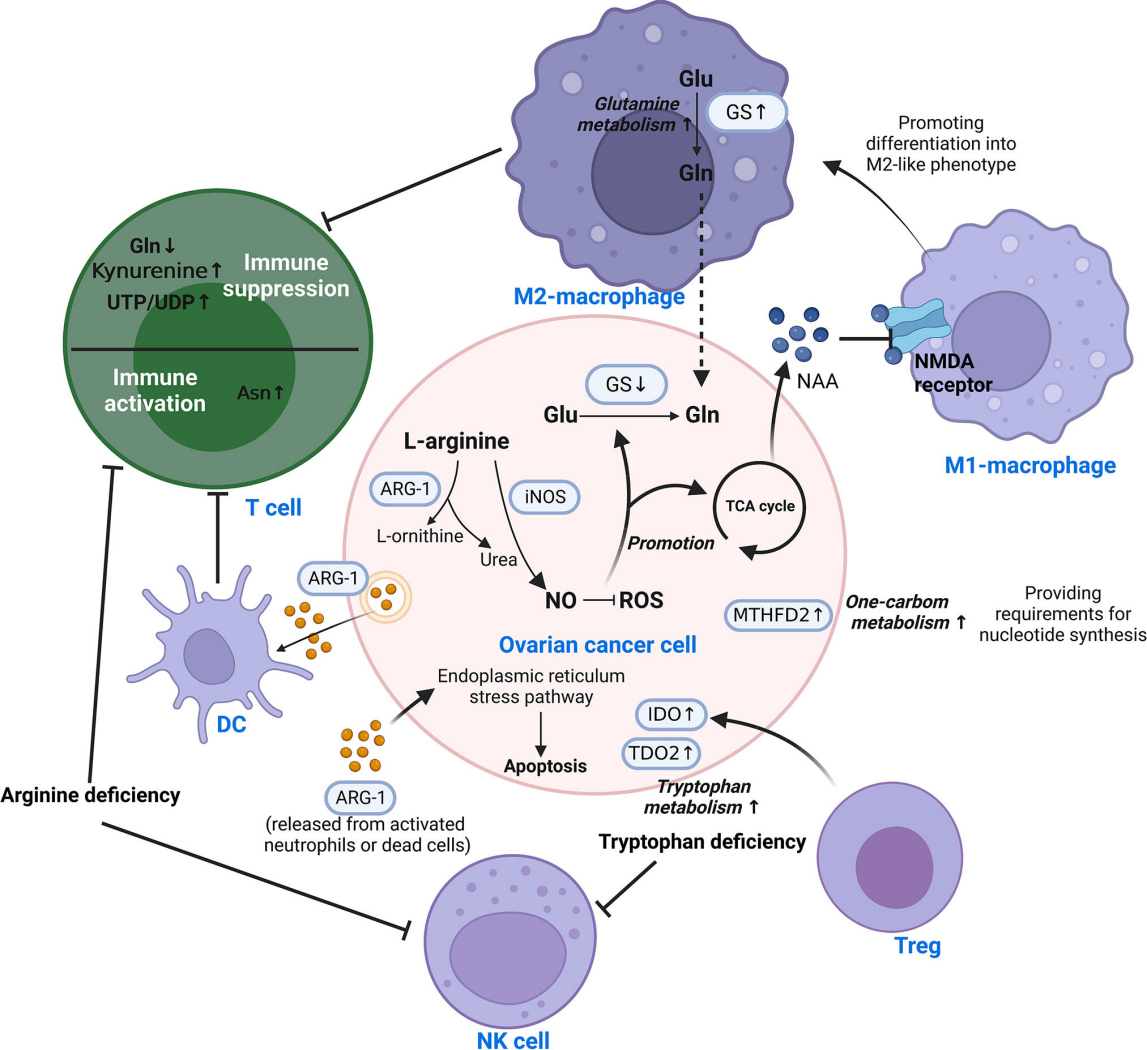
Reprogramming of amino acid metabolism in the TIME of ovarian cancer. Amino acid metabolism of ovarian cancer cells and immune cells in TIME is complementarily enhanced by metabolic reprogramming, which is responsible for mediating tumor progression and immune escape. Different cells are interconnected by amino acid metabolites. This involves the reprogramming of glutamine, arginine, tryptophan, aspartate, and one-carbon metabolism. Asn, Asparagine; ARG-1, Arginase-1; DC, Dendritic cell; Glu, Glutamate; Gln, Glutamine; GS, Glutamine synthetase; IDO, Indoleamine 2,3-dioxygenase; iNOS/NOS2, Nitric oxide synthase-2; ROS, Reactive oxygen species; TCA, Tricarboxylic acid; TDO2, tryptophan 2,3-dioxygenase; Teff, effector T cell; Treg, Regulatory T cell; MTHFD2, Methylenetetrahydrofolate dehydrogenase 2; NAA, N-acetylaspartate; NAMD receptor, N-methyl-D-aspartate receptor; NK, Natural killer; NO, nitric oxide; UDP, Uridine Diphosphate; UTP, Uridine Triphosphate.

The metabolism of amino acids has also been dramatically altered in ovarian cancer TIME to accommodate rapid growth. **Figure 3** illustrates how amino acid metabolites connect various cells. This entire process entails reprogramming glutamine, arginine, tryptophan, aspartate, and one-carbon metabolism. The details are discussed as follows.

#### 3.3.1 Glutamine

Glutamine is a significant nutrient source for the development of tumor cells (96). Its metabolism is significantly associated with the aggressiveness of OC, where glutamine synthetase (GS) is silenced in ovarian cancer cells in favor of extracellular glutamine addiction (96, 97). In highly invasive ovarian cancer, macrophages were found to be driven toward the M2-like subtype by glutamine metabolism (98). N-acetylaspartate (NAA) is secreted by ovarian cancer cells as an antagonist to suppress NMDA receptors on macrophages, causing macrophages to assume an M2-like phenotype with increased GS expression (98). Malignant ascites in ovarian cancer patients have reduced glucose uptake, decreased mitochondrial activity and downregulated glutamine carrier abundance in T cells (99, 100). This leads to poor T cell mitochondrial function and evasion of immunity under low glucose conditions.

#### 3.3.2 Arginine

Arginase converts L-arginine to L-ornithine and urea, which is known as the urea cycle. In the early stages of ovarian cancer, arginine metabolism is essential for the activation of T cells and control of immunological responses. In animal experiments, it was discovered that ovarian cancer cells express and release extracellular vesicles (EVs) containing ARG-1, which are taken up by DCs and prevent the proliferation of T cells (101). In contrast, ARG-1 released from activated neutrophils or dead cells induced apoptosis of cancer cells *via* the endoplasmic reticulum stress pathway (102). High amounts of arginine-1 boosted arginine metabolism in Tim-4^+^ TAMs (refilled from circulating monocytes), which in turn improved mitochondrial phagocytic activity in TAMs and ultimately inhibited T cell function (103). So do MDSCs. Strong arginine-1 expression and the production of ROS by MDSCs in human and mouse peritoneal ovarian cancers contribute to the immunosuppression of T cells (51). Arginine deficiency not only causes T cell malfunction but also decreases NK cell survival and cytotoxicity. *In vitro* experiments demonstrated that low L-Arg concentrations reduced the expression of activating receptors, NKp46 and NKp30, as well as the development of the NK zeta chain and the generation of IFN-gamma in NK-92 cell lines (104).

The production of nitric oxide (NO) by nitric oxide synthase is another catabolic mode of arginine. By raising NADPH and glutathione levels, NO lowers ROS levels and promotes glutamine and TCA cycling in ovarian cancer cells (105). Th17 cell growth is aided by physiological NO concentrations produced by MDSCs from ovarian cancer patients (106). Nitric oxide synthase 1 (NOS1) modulates S-nitrosylation at Cys351 of PFKM, which contributes to the reprogramming of glucose metabolism in ovarian cancer cells (107). Nitric oxide synthase-2 (NOS2/iNOS) expressed by endogenous T cells and NO generated by T cells are required for *de novo* Th17 differentiation from naive precursors and for induction of the Th17 phenotype in memory cells (106).

#### 3.3.3 Tryptophan

Tryptophan is an essential amino acid that can be catabolized or used to make tissue proteins. Patients with ovarian cancer showed accelerated tryptophan breakdown (108). There is evidence that in ovarian cancer, the enzyme indoleamine 2,3-dioxygenase (IDO) catalyzes the breakdown of tryptophan through the kynurenine pathway, resulting in immunosuppressive compounds that accelerate the growth of tumor cells and reduce antitumor immunity (109, 110). Specifically, it reduces tryptophan availability in immune cells, resulting in immunological escape by inhibiting the recruitment of NK cells and the infiltration of T cells (110, 111). Tregs can enhance IDO expression in ovarian cancer cells and synergize with hypoxia to extend the aggressiveness of OC (112). Meanwhile, tryptophan 2,3-dioxygenase (TDO2), the rate-limiting enzyme of the kynurenine pathway, was found to be upregulated in ovarian cancer tissues, which promotes tumor cell proliferation, migration and invasion (113).

#### 3.3.4 Aspartic acid and asparagine

Asparagine (Asn) is a metabolic byproduct that is released into the extracellular compartment by tumor cells in large amounts. A metabolomics-based study of EOC serum metabolites found that Asn may be an important factor influencing the pathogenesis of ovarian cancer (114). However, specific mechanisms have yet to be explored. The presence of Asn in the microenvironment dramatically enhances the activation, proliferation and tumor killing ability of CD8^+^ T cells both *in vivo* and *in vitro* (115).

#### 3.3.5 One-carbon metabolism

Large quantities of pyrimidines, thymidines, S-adenosylmethionine, and glutathione are necessary for rapid tumor proliferation for the synthesis of nucleotides. One-carbon (1C) metabolism backs up these substances required by tumors (116, 117). By affecting T cell functions, the folate-coupled metabolic enzyme methylenetetrahydrofolate dehydrogenase 2 (MTHFD2) serves as a crucial metabolic checkpoint in the 1C metabolic pathway (118). As a nicotinamide adenine dinucleotide (NAD^+^)-dependent enzyme with a considerable level of expression in ovarian cancer tissues, MTHFD2 positively correlates with both malignancy and prognosis (119). The synthesis of uridine-related metabolites, including UTP/UDP, is enhanced in the TIME by MTHFD2-mediated high levels of folate metabolism (120). It consequently stimulates PD-L1 transcription for the purpose of immune evasion (120). Furthermore, in IC metabolism, the methionine cycle is a major methyl donor, which is required for protein and nucleic acid methylation. Research has revealed the important role of methionine metabolism in T cell proliferation and differentiation (121). Cysteine is another significant metabolite in IC metabolism. It was discovered that ovarian cancer enhances tolerance to hypoxia through cysteine-mediated reprogramming of sulfur and carbon metabolism (122).

### 3.4 Metabolic signaling pathways in TIME reprogramming

The signaling pathways are linked to the shift of the metabolic landscape in ovarian cancer. Cancer cells exhibit persistent proliferative signals, and metabolic reprogramming facilitates this behavior.

The PI3K/Akt signaling pathway stimulates gluconeogenesis and lipid metabolism and activates tumor metabolism (123, 124). Salt-inducible kinase 2 (SIK2) overexpression in ovarian cancer cells activates the PI3K/AKT/HIF-1α pathway, upregulates HIF-1α expression, directly upregulates the transcription of major glycolytic genes and promotes glycolysis (123). SIK2 can promote mitochondrial fission and inhibit mitochondrial oxidative phosphorylation through phosphorylation of Drp1 at the Ser616 site (123). Moreover, SIK2 activates the PI3K/Akt signaling pathway and upregulates sterol regulatory element binding protein 1c (SREBP1c) and sterol regulatory element binding protein 2 (SREBP2), thus promoting the transcription of lipase FASN and cholesterol synthase HMGCR (124). This process improved the synthesis of cholesterol and FAs in ovarian cancer cells (124). In addition, ovarian cancer cells are capable of producing several forms of laminins (125). The Akt and MEK signaling pathways are activated in DCs cultivated with laminins *in vitro*, and a shift in the metabolic state of DCs induces the development of their reprogramming from bone marrow precursors to a suppressive phenotype (125).

The metabolic reprogramming of tumors is strongly correlated with the PI3K/Akt/mTOR pathway, which regulates cell growth and metabolism. The protein kinase mTOR is a serine/threonine enzyme that is a component of the mTORC1 and mTORC2 signaling complexes. In ovarian cancer, mTORC1 regulates glucose metabolism during CD8^+^ Treg differentiation by modulating HIF1α expression (126). Research has revealed that inhibiting GLScan synergize with the therapeutic effect of mTOR inhibitors on ovarian cancer (127).

Additionally, an aberrant MAPK signaling pathway is inextricably tied to metabolic reprogramming in ovarian cancer. It was found that TGF-β1 secreted by ovarian cancer cells could induce CD8+ Tregs through the p38 MAPK signaling pathway (128). In ovarian cancer, the widespread degradation of HIF-1 is encouraged by the silencing of TRPM7, which possesses ion channel and kinase activity. This promotes AMPK activation and changes glycolysis into oxidative phosphorylation (129).

As previously discussed, the Wingless (Wnt)/β-catenin pathway is hypothesized to be a driver of altered glycolysis, glutaminolysis, and lipogenesis (130). In ovarian cancer, hexokinase 2 increases CyclinD1/c-myc through the Wnt/β-catenin pathway, which in turn enhances oncogenesis and proliferation (131). In addition, macrophages that support ovarian cancer metastasis are linked to β-catenin expression (132). Inhibition of the Wnt/β-catenin pathway decelerates ovarian cancer progression and regulates the TIME by increasing cytotoxic T cell infiltration and decreasing MDSC infiltration (133).

## 4 Immunotherapeutic strategies targeting TIME metabolic reprogramming in ovarian cancer

Not only does metabolic adaptation of tumors take place throughout the course of OC, but it also happens during the course of therapy, which results in drug resistance (134–137). Metabolic reprogramming of the TIME in ovarian cancer leads to chemoresistance. M2-TAMs have a higher glucose uptake and utilization capacity, which stimulate the O-GlcNAcylation of lysosomal Cathepsin B (138). And it has been demonstrated that M2-TAMs could promote cancer metastasis and chemoresistance by increasing the activity of the hexosamine biosynthesis pathway (138). High levels of G6PD and glutathione-producing oxidoreductase are favorably associated with cisplatin resistance in OC (135). Moreover, paclitaxel resistance in ovarian cancer is associated with choline metabolism reprogramming. The expression of glycerophosphocholine phosphodiesterase 1 and glycerophosphodiester phosphodiesterase 1 in EOC was discovered to be elevated using proton magnetic resonance spectroscopy, and total choline was found to be elevated (136). Owing to the cunning metabolic adaptations of OC, monotherapy is frequently ineffective. For instance, when exposed to IDO1 inhibitors, ovarian cancer cells develop a metabolic adaptation that switches tryptophan catabolism to the 5-hydroxytryptamine pathway (137). NAD^+^ is increased as a result, which reduces T cell function and proliferation (137). The extracellular vesicles secreted by ovarian cancer can confer carboplatin resistance to tumor cells through hypoxia-induced metabolic dysregulation (mainly glycolysis and FAO) (139). Based on the metabolic adaptations described above in ovarian cancer, it is essential to take action to inhibit metabolic reprogramming during the treatment.

Immunotherapy allows the restoration of the T cell antitumor immune response through targeted metabolic reprogramming. Previously, we outlined the pertinent medications that target the metabolism of ovarian cancer as well as their clinical trials (29). Since there is an overlap of agents for the immunosuppressive microenvironment in ovarian cancer, we focused on immunotherapeutic strategies related to metabolic reprogramming targeting the TIME. **Table 2** summarizes the immunotherapy therapies and medicines listed herein for TIME in ovarian cancer.

**Table 2:** Immunotherapy targeting the metabolism of immune cells in ovarian cancer.

Immunotherapy approaches	Agents	Targets	Functions	Stage/Phase	Refs
Supplementation or deprivation of metabolism					
Metabolites supplementation	L-asparaginase	Depletion of asparagine	Cytotoxicity	Preclinical study	(140)
Metabolites supplementation	Glutamine	Supplement of glutamine	Synergistic enhancement of the antitumor effect of paclitaxel	Preclinical study	(141)
Metabolic deprivation through inhibition of glycolysis	2-deoxyglucose	hexokinase	Cytotoxicity, reducing lactate production, and blocking the expression of drug resistance-associated proteins.	Preclinical study	(30, 142)
Arginine deprivation	HuArgI (Co)-PEG5000	Human recombinant arginase I	Cytotoxicity and influencing viability and adhesion	Preclinical study	(143)
Allogeneic NK cell plus DCs and cytokines	6B11-OCIK	Autologous T cells plus dendritic cells and cytokines	Enhancement the function of immune system, rebuilding anti-tumor specific immunity and increasing the immune response of T cells	Phase I	(144)
Immune checkpoint inhibitors					
IDO and kynurenine pathways	CH223191	AHR antagonist	Reduction of AHR-induced PD-1 expression in T cells	Preclinical study	(145)
Combined therapy	GDC-0919 combined atezolizumab	IDO inhibitor and PD-L1 inhibitor	No significant therapeutic effect	Phase I	(146)
Combined therapy	An isotype of anti-PD-L1 antibody combined 968	PD-L1 inhibitors combined GLS inhibitor	Enhancement of T cell response and synergistic anti-tumor effects	Preclinical study	(147)
Others					
Autologous DC vaccination	DCVAC	An autologous DC-based vaccine	Improving progression-free survival in EOC	Phase II	(148, 149)
Autologous DC vaccination	Th17-induced FRα-loaded autologous DC vaccine	DCs programmed to induce a Th17 response to the OC antigen FRα	Inducingantigen-specific immunity and prolonging remission	Phase I	(150)
Oncolytic virotherapy	SV. IL12 in combination with anti-OX40	Inducing OX40 expression on CD4^+^ T cells	Significantly altering the transcriptome profile and metabolic program of T cells and activating T cell activity	Preclinical study	(151)

AHR, Aryl hydrocarbon receptor; DC, Dendritic cell; EOC, Epithelial ovarian cancer; FR, folate receptor; GLS, glutaminase; IDO, Indoleamine 2,3-dioxygenase; IL, Interleukin; OC, ovarian cancer; PD-L1, Programmed death ligand-1; Th17, IL-17-producing T helper; NK, Natural killer.

### 4.1 Supplementation or deprivation of metabolism

Certain antitumor effects can be achieved by directly supplementing metabolites. For example, L-asparaginase is a potential therapeutic enzyme. When cocultured with OVCAR-8 ovarian cancer cells, asparagine is rapidly depleted both inside and outside the cells, converting it to aspartic acid and assisting in tumor death (140). However, supplementation with amino acids *in vitro* shows no discernible benefit against malignancies. However, a potential involvement in disease recurrence and metastasis cannot be excluded. *In vivo* experiments on ovarian cancer-bearing mice showed that intraperitoneal injection of glutamine could enhance immune function and synergistically enhance paclitaxel’s antitumor effects (141).

On the other hand, metabolic deprivation is a form of starvation therapy for the treatment of ovarian cancer. The use of the specific glycolysis inhibitor 2-deoxyglucose triggers caspase-dependent apoptosis, reduces lactate production, and blocks the expression of resistance-associated proteins under low glucose conditions (30). AMPK activators can imitate the cytotoxicity induced by glucose starvation (142). Through arginine deprivation, human recombinant arginase I [HuArgI (Co)-PEG5000] can trigger autophagy and influence the motility and adhesion of ovarian cancer cells (143). Yu, J., et al. also discovered that simultaneous dual deprivation of lactate and glucose improved the antitumor effect (152). Furthermore, dietary changes might be beneficial in cancer prevention and treatment. A low-fat, high-fiber diet can help in the prevention and treatment of ovarian cancer (153, 154).

However, this adjustment of metabolic endpoint levels is extremely restricted, and most investigations have revealed no meaningful contribution to anticancer therapy in OC. It only works in a few ovarian cancer subtypes. For example, galactose intake may play a role in the development of borderline ovarian cancer in women who carry the galactose-1-phosphate uridyl transferase N314D polymorphism (155). Due to its accessibility and convenience, even though its therapeutic effect is modest, it is worthwhile to continue researching.

Adoptive cell transfer therapy (ACT) is a popular field of immunotherapy research. ACT is the collection of the patient’s own immune cells, followed by identification and *in vitro* cultivation to increase their quantity or improve their capacity for targeted killing (156). It can be helpful to improve the immune escape of ovarian cancer, especially for patients with recurrent ovarian cancer. An adoptive cell therapy of autologous T cells induced by a humanized anti-adiotypic antibody 6B11 minibody plus DCs and cytokines demonstrated preliminary safety and potential clinical efficacy in a phase I clinical trial of platinum-resistant recurrent or refractory ovarian cancer (144). A phase II study of allogeneic NK cell therapy for recurrent ovarian cancer showed suboptimal efficacy, and Treg cells were revealed to represent a treatment barrier (157). Allogeneic NK cell infusion in the most recent phase 1 clinical trial is still recruiting participants (158). In ovarian cancer TIME, the self-generated nanosystem known as KT-NE (KIRA6 loaded α-Tocopherol nanoemulsion) dramatically reduced lipid buildup in DCs and restored their immunological activity (159). In tumor-bearing mice, adoptive transfer of KT-NE-treated ID8-DCs increased host progression-free survival (159). Furthermore, PD-1 immunotherapy and KT-NE had synergistic effects (159). Additional clinical studies are needed to explore the therapeutic effects of ACT in ovarian cancer.

### 4.2 Immune checkpoint inhibitors

Checkpoint receptors on the surface of T cells, such as PD-1 and cytotoxic T lymphocyte-associated antigen 4 (CTLA-4), can suppress energy and metabolic changes in T cells and mediate immunosuppression when activated by the corresponding ligands (160). Immune checkpoint blockade (ICB) therapy enhances the tumor infiltration and effector functions of T cells by reprogramming metabolism. Classical PD-1, PD-L1, and CTLA-4 inhibitors have made great progress in the treatment of ovarian cancer. Immune checkpoints show a positive modulatory effect on metabolism (160, 161). PD-1 and CTLA-4 inhibitors can modulate the amount and activity of ARG-1, which prevents MDSCs from suppressing the immune system in ovarian cancer (161).

The IDO and kynurenine pathways are emerging metabolic checkpoints that promote T cell proliferation by preventing the synthesis of kynurenine (162). The aryl hydrocarbon receptor (AHR) antagonist CH223191 significantly decreased AHR-induced PD-1 expression in T cells (145).

However, the efficacy of ICB in the treatment of ovarian cancer is not obvious (163). In patients with advanced malignancies, combinations of PD-L1 inhibitors and IDO inhibitors have demonstrated acceptable safety and resistance (146). But there is no proof to back up the benefits of the combination. The metabolic reprogramming of the TIME is probably an important cause for the weak efficacy of ICB. The combination of drugs targeting metabolic reprogramming and ICIs is expected to improve the efficacy of ICB. Based on the glutamine dependence of ovarian cancer, the GLS inhibitor 968 increased the infiltration of CD3^+^ T cells and enhanced the apoptosis-inducing ability of cancer cells by CD8^+^ T cells. Combination with PD-L1 blockade enhanced the immune response to ovarian cancer (147).

The in-depth exploration of metabolic reprogramming has allowed for the continuous refinement of immunotherapy in ovarian cancer. For example, ICB induces not only IDO1 but also interleukin-4-induced-1 (IL4I1). The failure of clinical studies of ICB combined with IDO1 inhibition may be due to the presence of IL4I1 (164). The discovery of new metabolic immune checkpoints opens up a new path for cancer therapy.

### 4.3 Others

By targeting intracellular metabolic reprogramming, the metabolic state of T cells was induced to change, resulting in a more effective ovarian cancer treatment. Specific metabolism-related cell surface receptors can be identified as targets for selective delivery therapies based on the metabolic reprogramming properties of ovarian cancer. Folate receptor (FR) β in one-carbon metabolism can also be used as a regulatory target. It is expressed on activated macrophages in patients with EOC, and its blockade can inhibit nucleotide production, leading to significant immunotherapeutic effects (165, 166).

As more is known about tumor immunity, cancer vaccines have received increasing attention. Various cancer vaccines targeting immune cells have demonstrated a promising therapeutic landscape in ovarian cancer. In a phase II clinical trial, an autologous DC-based vaccination (DCVAC) significantly increased progression-free survival, and peripheral blood analysis revealed that DCVAC increases anti-cancer immunity in ovarian cancer patients with cool tumor nature (148, 149). A single-arm open-label phase I clinical trial indicates the safety of a Th17-induced FR-loaded autologous DC vaccination in inducing antigen-specific immunity and prolonging remission in ovarian cancer patients (150). Oncolytic virotherapy is an emerging immunotherapy. Oncolytic viruses invade tumor cells through cell surface molecules, infect and kill tumor cells, activate the immune response, and thus exert their tumor-killing effect (167). Lysozyme virus has high selectivity to tumor cells and high immune response intensity, thus maximizing the effect of immunotherapy. Gene set enrichment analysis showed that SV. IL12 in combination with anti-OX40 increased intracellular glycolysis and OXPHOS in T cells at the genetic level (151). Hulin-Curtis, S. L., et al. designed a phage peptide that binds to FRα on SKOV3 cell lines based on the selective high expression of FRα on ovarian cancer cells (168). It binds specifically to FRα, however, due to defective intracellular transport, it cannot yet be effectively targeted through FRα (168).

The above researches show promising therapeutic effects of tumor vaccines and oncolytic virotherapy for the treatment of ovarian cancer. However, because the relevant technology and exploration are not yet very mature, most of the current development is still only at the preclinical stage. More immunotherapy targeting TIME need to be developed in the future.

## 5 Perspectives and prospects

New insights suggest that tumors are not only a genetic disease but also highly associated with a suppressive immune microenvironment (16). Cellular metabolism is the key link between the extracellular environment and intracellular processes. Therefore, it is essential to explore the immunity and metabolism of ovarian cancer. By exploring the immunosuppressive microenvironment of ovarian cancer, we decipher what significant role environmental factors, especially immune cells, play in tumorigenesis, development, treatment and prognosis. Single-cell sequencing and integrated bioinformatics analysis have led to a greater understanding of ovarian cancer. J. Sun et al. described the immunogenomic landscape of HGSOC and identified immunological subtypes appropriate for immunotherapy (169). The application of immunogenomics, immunogenomics and molecular typing in ovarian cancer will contribute to subsequent targeted therapies. By deeply researching different immune subtypes, the application of precision therapy in ovarian cancer can be further expanded.

Ovarian cancer patients inevitably develop resistance to drugs, leading to treatment failure, which is the leading cause of death. Metabolic reprogramming of tumors occurs not only in tumorigenesis, progression, and metastasis, but different therapeutic measures also reshape tumor metabolism. It has been proven that surgery could increase the immune suppression of first-line ovarian cancer treatment (21). Metabolic reprogramming is extremely complex. In addition to the discussion in this review, epigenetics also plays an indelible role in the process of metabolic reprogramming (170). For example, ubiquitination and deubiquitination could alter cancer metabolism as one of the types of posttranslational modifications (171). Ovarian cancer restricts methyltransferase EZH2 expression in T cells by limiting aerobic glycolysis, thereby impairing T-cell-mediated antitumor immunity (172). Epigenetic regulation, in turn, can also affect cellular metabolism by modifying kinase activity. To illustrate, the DNA demethylating agent 5-aza-2-deoxycytidine reduces chemoresistance in cisplatin-resistant A2780cis cells and restores GS expression (173). Therefore, integrating the mechanism of interaction between metabolic reprogramming and epigenetics is also an important direction for future metabolic research in ovarian cancer, which could provide a theoretical foundation for treatment.

However, the metabolic phenotype of cancer is not invariant. Treatment resistance and metastases also cause metabolic reprogramming. It is feasible to increase the sensitivity of immunotherapy and chemotherapy as a new complement to the treatment of malignancies by targeting this feature. It is extremely promising to create medications to postpone the progression of tumors and to improve the sensitivity of cancer treatment based on the relevant theories. Therefore, it is crucial to continue researching metabolic reprogramming to enhance tumor immunotherapy.

## Author contributions

Conceptualization, YL and XL. Writing, review, and editing, YL and XtZ. Visualization, YL and YN. Supervision, XL and XZ. Funding acquisition, XZ and XL. All authors have read and agreed to the published version of the manuscript.

## Funding

This research was funded by the National Natural Science Foundation of China, grant number No. 81902662, the Natural Science Foundation of Sichuan Province, grant number No.2022NSFSC1531, the National Natural Science Foundation of China, grant number No. 81821002, Sichuan Science and Technology Program, grant number 2021YJ0011, and Foundation of Development and Related Diseases of Women and Children Key Laboratory of Sichuan Province, grant number No. 2022003. The APC was funded by West China Second Hospital.

## Acknowledgments

Figures in this review were created with BioRender.com.

## Conflict of interest

The authors declare that the research was conducted in the absence of any commercial or financial relationships that could be construed as a potential conflict of interest.

## Publisher’s note

All claims expressed in this article are solely those of the authors and do not necessarily represent those of their affiliated organizations, or those of the publisher, the editors and the reviewers. Any product that may be evaluated in this article, or claim that may be made by its manufacturer, is not guaranteed or endorsed by the publisher.

## Glossary

**Table:**

ACT	Adoptive cell transfer therapy
ADP	Adenosine diphosphate
AHR	Aryl hydrocarbon receptor
ARG1	Arginase-1
Asn	Asparagine
ATP	Adenosine triphosphate
AMPK	Adenosine monophosphate activated protein kinase
CTLA-4	Cytotoxic T lymphocyte-associated antigen 4
Cys	cysteine
CXCL	C-X-C motif chemokine ligand
DC	Dendritic cell
EOC	Epithelial ovarian cancer
EVs	Extracellular vesicles
FAs	Fatty acids
FAO	Fatty acid oxidation
FASN	Fatty acid synthase
FIGO	Federation of Gynecology and Obstetrics
FR	folate receptor
FSH	Follicle-stimulating hormone
GAPDH	Glyceraldehyde-3-phosphate dehydrogenase
GDE1	Glycerophosphodiester phosphodiesterase 1
GLS	glutaminase
GM-CSF	granulocyte-macrophage colony-stimulating factor
GPCPD1	Glycerophosphocholine phosphodiesterase 1
GS	glutamine synthetase
HIF-1α	Hypoxia-inducible factor-1
HGSOC	high-grade serous ovarian cancer
HMGCR	3-hydroxy-3-methylglutaryl-coenzyme A reductase
ICB	Immune checkpoint blockade
ICI	Immune checkpoint inhibitor
IDO	Indoleamine 2, 3-dioxygenase
IFN	Interferon
IL	Interleukin
IL4I1	Interleukin-4-induced-1
LPA	Lysophosphatidic acid
OC	ovarian cancer
OCCC	Ovarian clear cell carcinoma
OXPHOS	Oxidative phosphorylation
PDK1	Pyruvate dehydrogenase kinase 1
PDK2	Pyruvate dehydrogenase kinase 2
PD-1	Programmed death receptor 1
PD-L1	Programmed death ligand-1
PGE2	Prostaglandin E-2
PFKM	Phosphofructokinase-M
PI3K/Akt	Phosphatidylinositol 3-kinase/protein kinase B
PKM1	Pyruvate kinase M1
PKM2	Pyruvate kinase M2
PPAR	peroxisome proliferators-activated receptor
PPP	pentose phosphate pathway
Ser	Serine
SIK2	Salt-inducible kinase 2
SOCS3	cytokine signaling 3
SREBP	Sterol regulatory element binding protein
ROS	Reactive oxygen species
TAM	Tumor-associated macrophage
TCA	tricarboxylic acid
TDO2	tryptophan 2, 3-dioxygenase
Teff	effector T cell
TGF	transforming growth factor
Th17	IL-17-producing T helper
TIME	tumor immune microenvironment
Tmem	memory T cell
Treg	Regulatory T cell
TRPM7	Transient receptor potential melastatin 7L
MAPK	Mitogen-activated protein kinase
M-CSF	Macrophage colony-stimulating factor
MCT1	Monocarboxylate cotransporter 1
MDSCs	Myeloid-derived suppressor cells
MEK	methyl ethyl ketone
mRNA	Messenger ribose nucleic acid
MTHFD2	Methylenetetrahydrofolate dehydrogenase 2
mTOR	Mammalian target of rapamycin
NAA	N-acetylaspartate
NAD^+^	Nicotinamide adenine dinucleotide
NADPH	Nicotinamide adenine dinucleotide phosphate
NAMD receptor	N-methyl-D-aspartate receptor
NCCN	National Comprehensive Cancer Network
NK	Natural killer
NMDA receptor	N-methyl-D-aspartic acid receptor
NO	Nitric oxide
NOS1	Nitric oxide synthase 1
NOS2/iNOS	Nitric oxide synthase-2
UDP	Uridine Diphosphate
UTP	Uridine Triphosphate
VEGF	Vascular Endothelial Growth Factor
Wnt	Wingless
1C	One-carbon
